# A modular flow-chamber bioreactor concept as a tool for continuous 2D- and 3D-cell culture

**DOI:** 10.1186/1753-6561-7-S6-P87

**Published:** 2013-12-04

**Authors:** Christiane Goepfert, Grit Blume, Rebecca Faschian, Stefanie Meyer, Cedric Schirmer, Wiebke Müller-Wichards, Jörg Müller, Janine Fischer, Frank Feyerabend, Ralf Pörtner

**Affiliations:** 1 Institute of Bioprocess and Biosystems Engineering, Hamburg University of Technology Hamburg, D-21073, Germany; 2 Institute of Micro System Technology, Hamburg University of Technology, Hamburg, D-21073, Germany; 3 Department of Structural Research on Macromolecules, Institute of Materials Research, Helmholtz-Zentrum Geesthacht, Geesthacht, D-21502, Germany

## Background

Advanced cell culture models, especially long-term 3D systems, require bioreactors allowing for cultivation under continuous flow conditions. Such culture models are for example tissue engineered implants, 3D cultures for drug testing, *in vitro *models of cell growth and migration for wound healing studies, cell cultures for biomaterial testing. New challenges in drug testing and biomaterial development arise from regulatory requirements. Animal trials have to be replaced by cell culture assays, preferably by test systems with human material. Standard 2D monolayer cultures are often unsatisfactory and therefore tissue-like 3D cultures are suggested as an alternative. Here the design of a multi-well flow-chamber bioreactor as a tool for manufacturing advanced cell culture models is presented. Advantages of this reactor concept can be seen in constant flow conditions, removal of toxic reaction products, high cell densities, and improved metabolism [1]. The general design of the flow chamber bioreactor (FCBR) can easily be modified for different applications and analytical requirements.

## Concept

**Figure 1:**
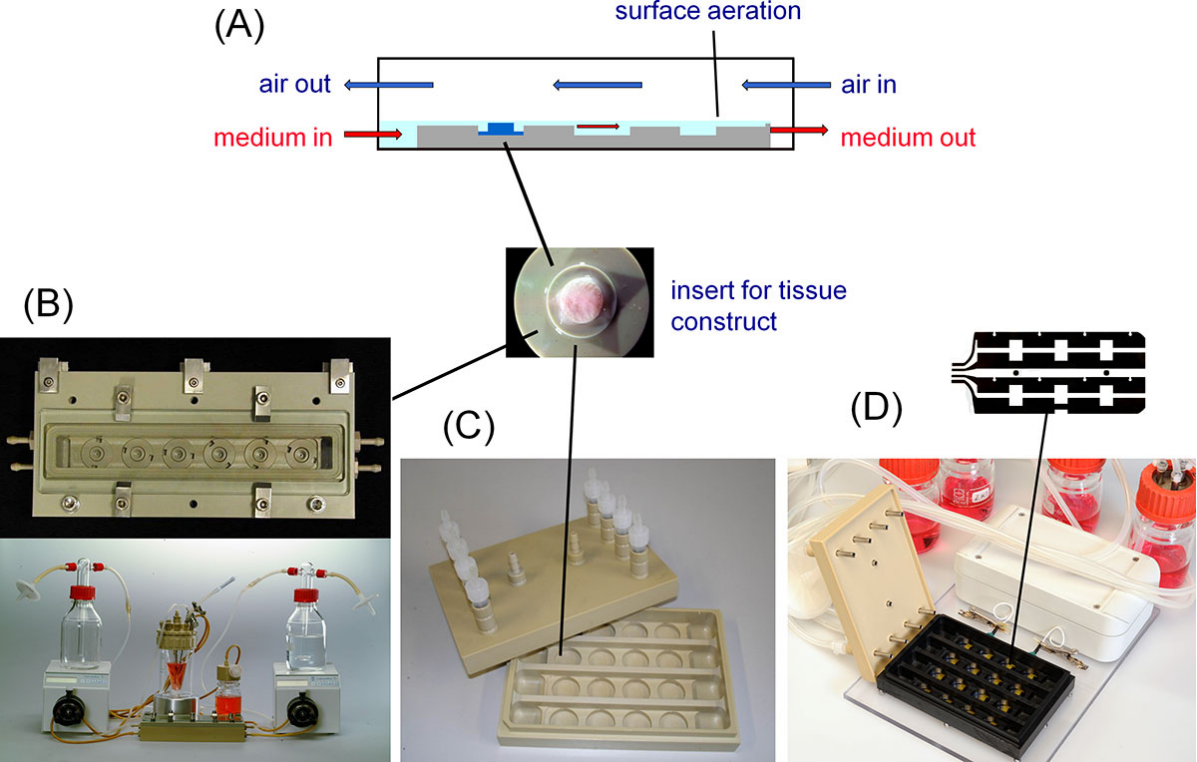
Flow-chamber bioreactor (FCBR, medorex, Germany)**(A) **Concept **(B) **Closed system (single channel) with aeration for tissue-engineered constructs **(C) **24 well plate-based modular bioreactor (medorex) for miniaturized constructs that permits the use of pipetting robots and standard plate readers **(D) **Flow chamber equipped with electrodes for stimulation.

The concept of the flow-chamber bioreactor (FCBR) comprises the following features (Figure 1A): Simultaneous cultivation of multiple tissue constructs in special inserts; oxygen supply via surface aeration directly in the chamber; a uniform and thin medium layer which is created by a small barrier at the end of the flow channel to minimize the diffusion distance from the gas phase to the tissue constructs; medium supply from a reservoir bottle in a circulation loop via peristaltic pumps.

Two designs are available: A closed system (single flow channel) with counter current flow of gas and medium for tissue-engineered constructs (Figure 1B), and a 24 well plate-based modular bioreactor (medorex, Nörten-Hardenberg, Germany) for miniaturized tissue constructs that permits the use of pipetting robots and standard plate readers (Figure 1C).

For the latter one, the design of the 4 channels can be customized for various applications (Table 1). The lid of the plate is connected to tubings for medium recirculation. Medium is supplied via the first well and removed from the last well of each row (Figure 1C). Therefore 4 wells per row are available for construct cultivation.

**Table 1:** Bioreactor configuration and applications

Bioreactor design		Potential applications	Example
I.	Single channel, 6 variable culture inserts for 3D scaffolds transparent cover plate active aeration	Long term cultivation of 3D tissue constructs under flow conditions, tissue cultivation on implantable biomaterials	Cultivation of cartilage-carrier constructs [2]

II.	4 flow channels for perfusion 24 well plate layout inserts for 3D scaffolds surface aeration gas supply from humidified incubator	Simultaneous cultivation of four 3D constructs per channel, 4 channels available, separate functional tests can be carried out on single constructs	3D cultures of liver cells [3], biomaterial testing

III.	As (II), transparent bottom plate for microscopy flow channels instead of separate wells	Cultivation of shear-responsive cells, integration of biomaterials possible (e.g. a collagen membrane)	Cultivation of sweat-gland associated cells (current)

IV.	As (II) plus integrated of electrodes for electrical stimulation and impedance measurement	Electrical stimulation of cell growth and orientation, impedance measurement of cell viability	Orientation of mitotic axis [5]

The closed system is aerated with humidified pre-mixed gas with optional composition. Therefore it can be handled independently from cell culture incubator. The 24 well-based system has to be placed in a humidified incubator for air supply from the incubator atmosphere.

## Fields of Application

For the above mentioned bioreactor designs, four applications are presented in the following.

**Example I: **The single flow-channel bioreactor (Figure 1 (B)) was designed for the generation of three-dimensional cartilage-carrier constructs [2]. The carriers consisting of a bone replacement material were covered with a 1-2 mm cartilage layer. This reactor was used for long-term cultivation of cartilage-carrier-constructs with improved biochemical parameters (e.g. content of glycosaminoclycan, collagen type II) under constant conditions.

**Example II: **The 24-well design was successfully applied to several cell culture models. Hepatocytes on porous 3D carriers were cultivated for 1-3 weeks and can be used as a model for drug testing [3]. After prolonged cultivation under continuous medium flow, the constructs are separated from each other for measurements in static operation mode to conduct viability and activity assays similar to procedures done in a standard multi well plate. Viability testing using Resazurin was performed repeatedly during cultivation. Furthermore, the EROD-assay for liver-specific cytochrome P450 activity was carried out at varying time points. Application for the resorption studies on magnesium implants is currently investigated by Prof. Willumeit, Dr. Feyerabend, HZ Geesthacht.

**Example III: **A third layout of the MWFB was realized with four parallel flow channels instead of the separate wells. There is also the possibility to carry out material tests for cell expansion on specific materials (e.g. polymer films, collagen membranes, different coatings etc.).

**Example IV: **Proliferation and migration of fibroblasts on collagen coated polymer foils integrated into the bioreactor was carried out using design IV (Figure 1 C). Electrical stimulation of NIH-3T3 fibroblasts resulted in the orientation of the cell cleavage plane perpendicular to the electric field vector. The electrodes were inserted into the chamber on a polymer foil clamped between the base plate and the 24 well plate equivalent top frame. The polymer foil can be removed and processed after the assays for staining and microscopic evaluation of the stimulated cells. The bottom plate was realized in a transparent material for microscopy. The frequency of unipolar pulses can be varied between 16 Hz and 2 kHz, the voltage between 0 up to 600 mV and stimulation pulse to pause ratios between 1:1, 1:10 and 1:100

## Conclusions

The flow chamber concept and its different modifications can be applied as an easily applicable and versatile tool for advanced cell culture models. The 24 well design is suitable for application in a standard cell culture lab without special bioreactor equipment: For medium supply, standard peristaltic pumps with 4 channels can be used. The bottom plate can be handled in a similar way as 24 well plates allowing for adaptation of standard assays to long-term 3D cultures, electrically stimulated cells, or primary cells cultivated on membranes consisting of various biomaterials.
